# Facile Synthesis, Characterization, and *In Vitro* Antimicrobial Screening of a New Series of 2,4,6-Trisubstituted-*s*-triazine Based Compounds

**DOI:** 10.1155/2015/571836

**Published:** 2015-01-31

**Authors:** Ravi Bhushan Singh, Nirupam Das, Md. Kamaruz Zaman

**Affiliations:** ^1^Institute of Pharmacy, Harish Chandra PG College, Varanasi 221 002, India; ^2^Department of Pharmaceutical Sciences, Assam University, Silchar 788 011, India; ^3^Department of Pharmaceutical Sciences, Dibrugarh University, Dibrugarh 786 004, India

## Abstract

A series of new 2,4,6-trisubstituted-*s*-triazine was synthesized, assessed for antimicrobial activity, and characterized by FTIR, ^1^HNMR, ^13^CNMR, and elemental analysis. The tested compounds, **4d**, **4g**, **4h**, **4k**, and **4n**, have shown considerable *in vitro* antibacterial efficacy with reference to the standard drug ciprofloxacin (MIC 3.125 *μ*gmL^−1^ against *B. subtilis*, *E. coli*, and *K. pneumoniae*). It was observed that compounds **4d** and **4h** displayed equipotent antibacterial efficacy against *B. subtilis* (MIC 3.125 *μ*gmL^−1^) and *S. aureus* (MIC 6.25 *μ*gmL^−1^). The studies demonstrated that the *para*-fluorophenylpiperazine substituted *s*-triazine (**4n**) was potent and exhibited broad spectrum antibacterial activity against *S. epidermidis*, *K. pneumoniae*, and *P. aeruginosa* with MIC of 6.25 *μ*gmL^−1^ and for *E. coli*, it showed an MIC of 3.125 *μ*gmL^−1^ equipotent with reference to the standard drug. Among all the compounds under investigation, compound **4g** also demonstrated significant antifungal activity (3.125 *μ*gmL^−1^) against *C. albicans*.

## 1. Introduction

Over the past decades, the development of new antibiotics has plummeted while antimicrobial resistance (AMR) has increased. Only a limited number of new antimicrobials have been developed in the last decade [1] and overtreatment with the available antibiotics has led to the emergence of AMR [2]. Despite big advances in antimicrobial therapies and strategies in counteracting infections, the emergence of AMR represents an emergency situation [3]. A high percentage of hospital-acquired infections are caused by highly resistant bacteria such as methicillin-resistant* Staphylococcus aureus* and vancomycin-resistant enterococci [4]. Further, infections caused by resistant microorganisms often fail to respond to the conventional treatment, resulting in prolonged illness and higher death risk. Considering the scenario, the World Health Organization has necessitated an urgent and consolidated effort to avoid regressing to the preantibiotic era [5]. Therefore, discovery of novel antimicrobial molecules and their rational use are crucial to combat microbial infections. In recent years, triazine derivatives have gained particular interest on account of their broad biological activities.

Triazine based compounds are proven to possess potent anticancer [6], antiretroviral [7], antimalarial [8], and antitubercular activity [9]. Additionally, it has also been reported that certain* s*-triazine derivatives possess potent antimicrobial activity [10–12] and have been explored by researchers successfully to yield potent antimicrobial agents. For example, compounds having thiazole nucleus attached with this structural motif displayed promising antifungal activity [13]. Furthermore, we have reported certain* s*-triazine derivative substituted with diethylamino side chain and* para*-methoxyphenyl ring that have demonstrated broad spectrum antibacterial activity comparable to streptomycin [14]. It is noteworthy that several* s*-triazines coupled with piperazine have shown promising antimicrobial activity. In particular, 1-(2-pyridyl) piperazine attached to the* s*-triazine nucleus containing 8-hydroxy quinoline in addition to 4-amino benzonitrile linkage showed potent activity against* Escherichia coli* compared to other analogues [15].

Recently, 4-benzyl piperazine derivatives of* s*-triazine were also found to be active against some Gram positive and Gram negative bacterial strains [16]. The importance of piperazine moiety has been substantiated by the fact that several* N*-alkyl and* N*-aryl piperazine derivatives showed potent antibacterial activity against resistant microbial strains [17, 18]. Additionally, thiosemicarbazones of piperazine bearing arylmethylidene also displayed potent antimicrobial activity compared to ciprofloxacin [19]. Thus considering the above factors and with a goal to explore and improve the antimicrobial profile of* s*-triazines, we carried out the synthesis of a series of new 2,4,6-trisubstituted-*s*-triazines. All the compounds are sequentially substituted with aryl, diethylamino, and/or morpholinyl substituent at 2nd, 4th, and 6th position, respectively, of* s*-triazine. In addition, the compounds are invariably substituted with piperazine,* N*-methylpiperazine or aryl substituted piperazine as one of its substituents of* s*-triazine scaffold. The precursor for the compounds is cyanuric chloride and the synthetic route involves temperature controlled nucleophilic substitution reaction. The reaction relies on differential reactivity of the substituted chlorine atoms and the easy displacement of the chloride with nucleophiles for nonsymmetrical trisubstituted functionalization [20]. The newly synthesized compounds were screened for their possible antimicrobial activity to identify new candidates as antibacterial and antifungal agents.

## 2. Materials and Methods

All the chemicals used in this experiment were of analytical grade and procured from Merck (India) and Hi-Media (India). Cyanuric chloride was obtained from Lonza Ltd., Switzerland, as a gift sample. Melting points were determined in open capillaries using Veego VMP1 melting point apparatus, Veego Instruments Corporation (Mumbai, India), and are uncorrected. The progress of the reactions was monitored by thin layer chromatography developed with n-hexane/diethyl ether (1 : 1) and performed on Merck silica gel 60 F254 aluminium sheets and products were purified by recrystallization. IR spectra were recorded on a Perkin Elmer-Spectrum RX-1 spectrophotometer, PerkinElmer, Inc. (California, USA), on potassium bromide pellets and were recorded in cm^−1^. ^1^H NMR and ^13^C NMR spectra were performed on a JEOL AL300 FT-NMR (300 MHz), Jeol Ltd., Japan, in DMSO-d_6_/CDCl_3_ using tetramethylsilane as internal standard and the chemical shifts are reported in ppm (*δ*). Elemental analysis (C, H, and N) were performed on Exeter CE-440 elemental analyzer (Exeter Analytical Inc., USA).

### 2.1. Chemistry

**Scheme 1:**
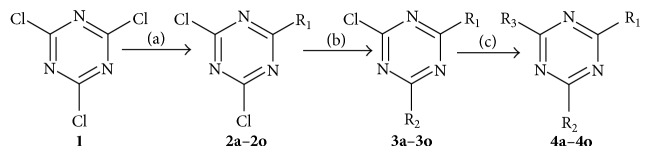
General scheme for the synthesis of compounds** 4a**–**4o**. Reagents and conditions: (a) R_1_H, acetone, stir, 0–5°C, 2 h, Na_2_CO_3_; (b) R_2_H, acetone, stir, r.t., 3 h, Na_2_CO_3_; (c) R_3_H, 1,4 dioxane, reflux, 60–65°C, 6–8 h.

Scheme 1 outlines the general classic three-step temperature controlled method of synthesis of 2,4,6-trisubstituted-*s*-triazine derivatives (Table 1) as mentioned in the literature [21].

### 2.2. Synthesis

#### 2.2.1. General Method of Synthesis of 2-Substituted-4,6-dichloro-*s*-triazine Derivatives (**2a**–**2o**)

Cyanuric chloride (**1**) (0.01 mol) was dissolved in acetone (25 mL) and different substituted nucleophiles (R_1_H) (Table 1) (0.02 mol) were added to it at 0–5°C and stirred for 2 h. Thereafter, sodium carbonate solution (10%) was added to neutralize the hydrochloric acid evolved during the reaction. The reaction mixture was then poured into crushed ice and the solid separated out was filtered, washed with water, and recrystallized from ethanol.

#### 2.2.2. General Method of Synthesis of 2,4-Disubstituted-6-chloro-*s*-triazine Derivatives (**3a**–**3o**)

2-Substituted-4,6-dichloro-*s*-triazine derivatives (**2a**–**2o**) were added to 100 mL of acetone and different substituted nucleophiles (R_2_H) (Table 1) (0.02 mol) were added to it and stirred at room temperature for 3 h. Sodium carbonate solution (10%) was added to neutralize the hydrochloric acidevolved during the reaction and the product was filtered and washed with cold water and recrystallized from ethanol.

#### 2.2.3. General Method of Synthesis of 2,4,6-Trisubstituted-*s*-triazine Derivatives (**4a**–**4o**)

2,4-Disubstituted-6-chloro-*s*-triazine derivatives** (3a**–**3o) **were added to 20 mL of 1,4–dioxane and different substituted nucleophiles (R_3_H) (0.02 mol) (Table 1) were refluxed at 60–65°C for 6–8 h during which anhydrous sodium carbonate (10%) was added in fractions (0.2 g) over 2 hr. The content was then poured into crushed ice; precipitate obtained was dried and recrystallized from ethanol to afford the target compounds (**4a**–**4o**).

#### 2.2.4. *N*2-(4-Methoxyphenyl)-*N*4-(4-methyl-2-nitrophenyl)-6-(piperazin-1-yl)-*s*-triazine-2,4-diamine (**4a**)

Pale brown solid, Yield: 80%, mp 155–157°C; IR cm^−1^ (KBr): 3315 (2° –NH stretch amines); 2965 (methyl CH asymmetric stretch); 2833 (methoxy –CH stretch); 1575 (–C=N ring stretch); 1331, 1523 (–NO stretch), 805 (CN stretch* s*-triazine). ^1^H NMR (300 MHz, DMSO-d_6_):  *δ*  1.98 (s, 1H, piperazinyl–NH),  *δ*  2.56 (s, 3H, –CH_3_),  *δ*  3.21 (t, 4H, piperazinyl),  *δ*  3.53 (t, 4H, piperazinyl),  *δ*  3.78 (s, 3H, –OCH_3_),  *δ*  6.45–7.71 (m, 7H, ArH),  *δ*  8.36 (s, 2H, –NH). ^13^C NMR (75 MHz, DMSO-d_6_):  *δ*  181.3 (C6* s*-triazine, C–N piperazinyl linkage), 173.1 (C2* s*-triazine, C–N 4-methoxyphenyl linkage), 157.5 (C4* s*-triazine, C–N 4-methyl-2-nitrophenyl linkage), 153.3, 137.5, 134.2, 129.8, 117.2 (12C aryl carbons), 56.4 (–OCH_3_), 51.2, 46.3 (4C piperazinyl carbons), 23.5 (*N*4 aryl 4–CH_3_). Anal. Calcd for: C_21_H_24_N_8_O_3_: C, 57.79; H, 5.54; N, 25.67; Found: C: 57.58; H: 5.55; N: 25.73.

#### 2.2.5. *N*-(4-Methyl-2-nitrophenyl)-4-morpholino-6-(piperazin-1-yl)-*s*-triazin-2-amine (**4b**)

Yellow solid, Yield: 86%, mp 160–162°C; IR cm^−1^ (KBr): 3321 (2° –NH stretch); 2966 (methyl CH asymmetric stretch); 1577 (–C=N ring str); 1334, 1527 (–NO stretch); 1117 (morpholinyl –C–O–C stretch); 809 (CN stretch* s*-triazine). ^1^H NMR (300 MHz, CDCl_3_):  *δ*  1.87 (s, 1H, piperazinyl –NH),  *δ*  2.62 (s, 3H, –CH_3_),  *δ*  3.18 (m, 4H, piperazinyl),  *δ*  3.47 (m, 4H, piperazinyl),  *δ*  3.65 (t, 4H, morpholinyl),  *δ*  3.81 (t, 4H, morpholinyl),  *δ*  6.60–7.74 (m, 3H, ArH),  *δ*  8.47 (s, 1H, –NH). ^13^C NMR (75 MHz, CDCl_3_):  *δ*  183.2 (C4* s*-triazine, C–N morpholino linkage), 179.3 (C6* s*-triazine, C–N piperazinyl linkage), 160.8 (C2* s*-triazine, C–N 4-methyl-2-nitrophenyl linkage), 139.4, 136.0, 127.1, 117.2 (6C aryl carbons), 65.2, 51.7 (4C morpholino carbons), 46.7, 44.9 (4C piperazinyl carbons), 23.1 (C2 aryl 4–CH_3_). Anal. Calcd for C_18_H_24_N_8_O_3_: C, 53.99; H, 6.04; N, 27.98; Found: C, 53.95; H, 6.02; N, 28.01.

#### 2.2.6. *N*2-(4-Methoxyphenyl)-*N*4-(4-methyl-2-nitrophenyl)-6-(4-methylpiperazin-1-yl)-*s*-triazine-2,4-diamine (**4c**)

Pale brown solid, Yield: 76%, mp 181–183°C; IR cm^−1^ (KBr): 3317 (2° –NH stretch); 2962 (methyl CH asymmetric stretch); 2831 (methoxy –CH stretch); 1570 (–C=N ring stretch); 1333, 1525 (–NO stretch), 805 (CN stretch* s*-triazine). ^1^H NMR (300 MHz, DMSO-d_6_):  *δ*  2.25 (s, 3H, –CH_3_),  *δ*  2.49 (s, 3H, –CH_3_),  *δ*  2.98 (t, 4H, piperazinyl),  *δ*  3.36 (t, 4H, piperazinyl),  *δ*  3.75 (t, 3H, –OCH_3_),  *δ*  6.37–7.78 (m, 7H, ArH),  *δ*  8.63 (s, 2H, NH). ^13^C NMR (75 MHz, DMSO-d_6_):  *δ*  180.6 (C6* s*-triazine, C–N piperazinyl linkage), 164.8 (C4* s*-triazine, C–N 4-methyl-2-nitrophenyl linkage), 158.9 (C2* s*-triazine, C–N 4-methoxyphenyl linkage), 138.5, 136.1, 134.5, 130.3, 124.9 117.1, 114.1 (12C aryl carbons), 56.7 (*N*2 aryl 4 –OCH_3_), 52.2, 47.7 (4C piperazinyl carbons), 42.5 (C6 piperazinyl 4–CH_3_), 23.4 (*N*4 aryl 4–CH_3_). Anal. Calcd for: C_22_H_26_N_8_O_3_: C, 58.65; H, 5.82; N, 24.87; Found: C: 57.48; H: 5.83; N: 24.85.

#### 2.2.7. *N*2-(4-Bromophenyl)-*N*4-(4-chlorophenyl)-6-(4-methylpiperazin-1-yl)-*s*-triazine-2,4-diamine (**4d**)

Pale brown solid, Yield: 78%, mp 171–173°C; IR cm^−1^ (KBr): 3321 (2° –NH stretch); 1575 (–C=N ring stretch); 810 (CN stretch* s*-triazine). ^1^H NMR (300 MHz, DMSO-d_6_):  *δ*  2.25 (s, 3H, –CH_3_),  *δ*  3.13 (t, 4H, piperazinyl), 3.31 (t, 4H, piperazinyl),  *δ*  6.35–7.74 (m, 8H, ArH),  *δ*  8.54 (s, 2H, NH). ^13^C NMR (75 MHz, DMSO-d_6_):  *δ*  181.2 (C6* s*-triazine, C–N piperazinyl linkage), 166.4 (C2* s*-triazine, C–N 4–bromophenyl linkage), 159.2 (C4* s*-triazine, C–N 4–chlorophenyl linkage), 139.3, 136.0, 135.4, 133.7, 130.3, 126.1 118.5, 116.9 (12C aryl carbons), 54.7, 45.8 (4C piperazinyl carbons), 43.1 (C6 piperazinyl 4–CH_3_). Anal. Calcd for: C_20_H_21_BrClN_7_: C, 50.59; H, 4.46; N, 20.65 Found: C: 50.44; H: 4.45; N: 20.60.

#### 2.2.8. *N*2-(4-Methoxyphenyl)-*N*4-(4-methyl-2-nitrophenyl)-6-(2-methylpiperazin-1-yl)-*s*-triazine-2,4-diamine (**4e**)

Pale brown solid, Yield: 82%, mp 210–212°C; IR cm^−1^ (KBr): 3315 (2° –NH stretch); 2965 (methyl CH asymmetric stretch); 2835 (methoxy –CH stretch); 1575 (–C=N ring stretch); 1334, 1530 (–N–O stretch); 805 (CN stretch* s*-triazine). ^1^H NMR (300 MHz, DMSO-d_6_):  *δ*  2.08 (s, 1H, –NH),  *δ*  2.20 (s, 3H, –CH_3_),  *δ*  2.55 (s, 3H, –CH_3_),  *δ*  2.79–3.23 (m, 7H, piperazinyl),  *δ*  3.73 (s, 3H, –OCH_3_),  *δ*  6.56–7.81 (m, 7H, Ar–H),  *δ*  8.37 (s, 2H, NH). ^13^C NMR (75 MHz, DMSO-d_6_):  *δ*  181.2 (C6* s*-triazine, C–N piperazinyl linkage), 166.7 (C4* s*-triazine, C–N 4-methyl-2-nitrophenyl linkage), 160.3 (C4* s*-triazine, C–N 4–methoxyphenyl linkage), 138.5, 136.6, 134.8, 135.2, 130.3, 124.9, 117.1, 115.1 (12C aryl carbons), 59.5, 57.6 (piperazinyl), 55.9 (*N*2 aryl 4 –OCH_3_), 50.4, 47.1 (piperazinyl), 23.4 (*N*4 aryl 4–CH_3_), 16.5 (C6 piperazinyl 2–CH_3_). Anal. Calcd for: C_22_H_26_N_8_O_3_: C, 58.65; H, 5.82; N, 24.87; Found: C: 58.63; H: 5.84; N: 24.85.

#### 2.2.9. *N*2-(4-Methoxyphenyl)-6-(2-methylpiperazin-1-yl)-*N*4-(4-nitrophenyl)-*s*-triazine-2,4-diamine (**4f**)

Brown solid, Yield: 82%, mp 207–209°C; IR cm^−1^ (KBr): 3318 (2° –NH stretch); 2830 (methoxy –CH stretch); 1570 (–C=N ring stretch); 1333, 1528 (NO stretch); 808 (CN stretch* s*-triazine). ^1^H NMR (300 MHz, DMSO-d_6_):  *δ*  1.98 (s, 1H, –NH),  *δ*  2.21 (s, 3H, –CH_3_),  *δ*  2.75–3.31 (m, 7H, piperazinyl),  *δ*  3.74 (s, 3H, –OCH_3_),  *δ*  6.36–7.93 (s, 8H, ArH),  *δ*  8.33 (s, 2H, NH). ^13^C NMR (75 MHz, DMSO-d_6_):  *δ*  180.1 (C6* s*-triazine, C–N piperazinyl linkage), 165.5 (C4* s*-triazine, C–N 4-nitrophenyl linkage), 158.1 (C2* s*-triazine, C–N 4–methoxyphenyl linkage), 149.2, 138.4, 134.6, 129.5, 121.8, 117.6 (12C aryl carbons), 61.4 (piperazinyl), 56.5 (*N*2 aryl 4 –OCH_3_), 51.7, 49.3, 46.2 (piperazinyl), 16.7 (C6 piperazinyl 2–CH_3_). Anal. Calcd for: C_21_H_24_N_8_O_3_: C, 57.79; H, 5.54; N, 25.67; Found: C: 57.92; H: 5.53; N: 25.59.

#### 2.2.10. *N*2-(4-Methoxyphenyl)-*N*4-(4-methyl-2-nitrophenyl)-6-(4-(4-nitrophenyl)piperazin-1-yl)-*s*-triazine-2,4-diamine (**4g**)

Pale yellow solid, Yield: 84%, mp 190–192°C; IR cm^−1^ (KBr): 3324 (2° –NH stretch); 2963 (methyl CH asymmetric stretch); 2837 (methoxy –CH stretch); 1577 (–C=N ring stretch); 1335, 1531 (NO stretch); 809 (CN stretch* s*-triazine). ^1^H NMR (300 MHz, DMSO-d_6_):  *δ*  2.37 (s, 3H, –CH_3_),  *δ*  3.22 (t, 4H, piperazinyl),  *δ*  3.61 (t, 4H, piperazinyl),  *δ*  3.75 (s, 3H, –OCH_3_),  *δ*  6.38–8.01 (s, 11H, ArH),  *δ*  8.36 (s, 2H, –NH). ^13^C NMR (75 MHz, DMSO-d_6_):  *δ*  179.8 (C6* s*-triazine, C–N piperazinyl linkage), 164.6 (C4* s*-triazine, C–N 4-methyl-2-nitrophenyl linkage), 160.7 (C2* s*-triazine, C–N 4–methoxyphenyl linkage), 156.5, 151.3, 137.8, 136.1, 134.9, 130.3, 124.6, 119.3, 116.2 (18C aryl carbons), 56.3 (*N*2 aryl 4 –OCH_3_), 50.9, 46.4 (4C piperazinyl carbons), 23.2 (*N*4 aryl 4–CH_3_). Anal. Calcd for: C_27_H_27_N_9_O_5_: C, 58.16; H, 4.88; N, 22.61; Found: C: 58.21; H: 4.87; N: 22.67.

#### 2.2.11. *N*2-(4-Methyl-2-nitrophenyl)-*N*4-(4-nitrophenyl)-6-(4-(4-nitrophenyl)piperazin-1-yl)-*s*-triazine-2,4-diamine (**4h**)

Pale yellow solid, Yield: 84%, mp 213–215°C; IR cm^−1^ (KBr): 3319 (2° –NH stretch); 2963 (methyl CH asymmetric stretch); 1575 (–C=N ring stretch); 1331, 1528 (NO stretch); 809 (CN stretch* s*-triazine). ^1^H NMR (75 MHz, DMSO-d_6_):  *δ*  2.37 (s, 3H, –CH_3_),  *δ*  3.24 (t, 4H, piperazinyl),  *δ*  3.45 (t, 4H, piperazinyl),  *δ*  6.65–8.18 (m, 11H, ArH),  *δ*  8.31 (s, 2H, –NH). ^13^C NMR (75 MHz, DMSO-d_6_):  *δ*  180.6 (C6* s*-triazine, C–N piperazinyl linkage), 167.9 (C2* s*-triazine, C–N 4-methyl-2-nitrophenyl linkage), 159.2 (C4* s*-triazine, C–N 4-nitrophenyl linkage), 156.4, 148.8, 136.5, 127.6, 122.0, 118.9 116.2 (18C aryl carbons), 49.6, 46.7 (4C piperazinyl carbons), 23.5 (*N*2 aryl 4–CH_3_). Anal. Calcd for: C_26_H_24_N_10_O_6_: C, 54.54; H, 4.23; N, 24.46; Found: C: 54.73; H: 4.22; N: 24.51.

#### 2.2.12. *N*-(4-Chlorophenyl)-4-morpholino-6-(4-(4-nitrophenyl)piperazin-1-yl)-*s*-triazine-2-amine (**4i**)

Pale yellow solid, Yield: 84%, mp 165–167°C; IR cm^−1^ (KBr): 3323 (2° –NH stretch); 1573 (–C=N ring stretch); 1119 (morpholinyl C–O–C stretch); 1336, 1527 (NO stretch); 810 (CN stretch* s*-triazine). ^1^H NMR (300 MHz, DMSO-d_6_):  *δ*  3.21 (t, 4H, piperazinyl),  *δ*  3.41 (t, 4H, piperazinyl),  *δ*  3.56 (t, 4H, morpholino),  *δ*  3.84 (t, 4H, morpholino),  *δ*  6.55–8.16 (m, 8H, ArH),  *δ*  8.48 (s, 1H, –NH). ^13^C NMR (75 MHz, DMSO-d_6_):  *δ*  182.4 (C6* s*-triazine, C–N piperazinyl linkage), 179.7 (C4* s*-triazine, C–N morpholino linkage), 165.2 (C2* s*-triazine, C–N 4-chlorophenyl linkage), 151.3, 146.2, 130.8, 126.5, 119.1, 115.2 (12C aryl carbons), 66.4 (morpholino), 50.7, 48.3 (4C piperazinyl carbons), 46.5 (morpholino). Anal. Calcd for: C_23_H_25_ClN_8_O_3_: C, 55.59; H, 5.07; N, 22.55; Found: C: 55.61; H: 5.18; N: 22.48.

#### 2.2.13. 6-(4-(4-Chlorophenyl)piperazin-1-yl)-*N*2-(4-methoxyphenyl)-*N*4-(4-methyl-2-nitrophenyl)-*s*-triazine-2,4-diamine (**4j**)

Yellow solid, Yield: 84%, mp 208–210°C; IR cm^−1^ (KBr): 3321 (2° –NH stretch); 2835 (methoxy –CH stretch); 2964 (methyl CH asymmetric stretch); 1577 (–C=N ring stretch); 1336, 1528 (NO stretch); 809 (CN stretch* s*-triazine). ^1^H NMR (300 MHz, DMSO-d_6_):  *δ*  2.42 (s, 3H, –CH_3_),  *δ*  3.18 (t, 4H, piperazinyl),  *δ*  3.51 (t, 4H, piperazinyl),  *δ*  3.73 (s, 3H, –OCH_3_),  *δ*  6.35–7.87 (m, 11H, ArH),  *δ*  8.43 (s, 2H, –NH).^13^C NMR (75 MHz, DMSO-d_6_):  *δ*  180.8 (C6* s*-triazine, C–N piperazinyl linkage), 165.6 (C4* s*-triazine, C–N 4-methyl-2-nitrophenyl linkage), 163.5 (C2* s*-triazine, C–N 4-methoxyphenyl linkage), 155.4, 138.0, 135.6, 134.9, 128.7, 117.2, 114.7 (18C aryl carbons), 56.9 (*N*2 aryl 4 –OCH_3_), 49.6, 46.2 (4C piperazinyl carbons), 23.5 (*N*4 aryl 4–CH_3_). Anal. Calcd for: C_27_H_27_ClN_8_O_3_: C, 59.28; H, 4.98; N, 20.48; Found: C: 59.19; H: 4.97; N: 20.61.

#### 2.2.14. 6-(4-(4-Chlorophenyl)piperazin-1-yl)-*N*2,*N*2-diethyl-*N*4-(4-nitrophenyl)-*s*-triazine-2,4-diamine (**4k**)

Yellow solid, Yield: 84%, mp 211–213°C; IR cm^−1^ (KBr): 3316 (2° –NH stretch); 1570 (–C=N ring stretch); 1337, 1528 (NO stretch); 1314 (3°CN stretch); 805 (CN stretch* s*-triazine). ^1^H NMR (300 MHz, DMSO-d_6_):  *δ*  2.23 (s, 4H, –CH_2_),  *δ*  3.18 (t, 4H, piperazinyl),  *δ*  3.33 (s, 6H, –CH_3_), 3.54 (t, 4H, piperazinyl),  *δ*  6.49–8.05 (s, 8H, ArH),  *δ*  8.52 (s, 1H, –NH). ^13^C NMR (75 MHz, DMSO-d_6_):  *δ*  181.5 (C6* s*-triazine, C–N piperazinyl linkage), 179.7 (C2* s*-triazine, C–N diethylamino linkage), 165.4 (C4* s*-triazine, C–N 4-nitrophenyl linkage), 151.8, 147.7, 145.2, 136.3, 130.8, 117.5, 115.2 (12C aryl carbons), 51.9, 49.4 (4C piperazinyl carbons), 44.6 (4C diethyl –CH_2_), 18.3 (6C diethyl –CH_3_). Anal. Calcd for: C_23_H_27_ClN_8_O_2_: C, 57.20; H, 5.63; N, 23.20; Found: C: 57.36; H: 5.61; N: 23.25.

#### 2.2.15. *N*-(4-Chlorophenyl)-4-(4-(4-chlorophenyl)piperazin-1-yl)-6-morpholino-*s*-triazin-2-amine (**4l**)

Pale yellow solid, Yield: 85%, mp 205–207°C; IR cm^−1^ (KBr): 3324 (2° –NH stretch); 1576 (–C=N ring stretch); 1118 (morpholinyl C–O–C stretch); 807 (CN stretch* s*-triazine). ^1^H NMR (300 MHz, DMSO-d_6_):  *δ*  3.19 (t, 4H, piperazinyl),  *δ*  3.44 (t, 4H, piperazinyl),  *δ*  3.53 (t, 4H, morpholinyl),  *δ*  3.80 (t, 4H, morpholinyl),  *δ*  6.65–7.72 (m, 8H, Ar–H), 8.43  *δ*  (s, 1H, –NH). ^13^C NMR (75 MHz, DMSO-d_6_):  *δ*  183.1 (C4* s*-triazine, C–N piperazinyl linkage), 178.3 (C6* s*-triazine, C–N morpholino linkage), 167.6 (C2* s*-triazine, C–N 4–chlorophenyl linkage), 148.2, 144.5, 130.5, 124.3, 118.2, 116.1 (12C aryl carbons), 65.3 (morpholino), 51.4, 49.2 (4C piperazinyl carbons), 46.9 (morpholino). Anal. Calcd for: C_23_H_25_Cl_2_N_7_O: C, 56.79; H, 5.18; N, 20.16; Found: C: 56.62; H: 5.20; N: 20.23.

#### 2.2.16. 6-(4-(4-Fluorophenyl)piperazin-1-yl)-*N*2-(4-methoxyphenyl)-*N*4-(4-methyl-2-nitrophenyl)-*s*-triazine-2,4-diamine (**4m**)

Yellow solid, Yield: 84%, mp 184–186°C; IR cm^−1^ (KBr): 3321 (2° –NH stretch); 2961 (methyl CH asymmetric stretch); 2830 (methoxy CH stretch); 1575 (–C=N ring stretch); 809 (CN stretch* s*-triazine). ^1^H NMR (300 MHz, DMSO-d_6_):  *δ*  2.49 (s, 3H, –CH_3_),  *δ*  3.16 (t, 4H, piperazinyl),  *δ*  3.55 (t, 4H, piperazinyl),  *δ*  3.71 (s, 3H, –OCH_3_),  *δ*  6.39–7.96 (m, 11H, ArH),  *δ*  8.41 (s, 2H, NH). ^13^C NMR (75 MHz, DMSO-d_6_):  *δ*  181.5 (C6* s*-triazine, C–N piperazinyl linkage), 166.8 (C4* s*-triazine, C–N 4-methyl-2-nitrophenyl linkage), 162.2 (C2* s*-triazine, C–N 4-methoxyphenyl linkage), 152.4, 137.3, 135.1, 133.4, 129.2, 116.3, 113.9 (18C aryl carbons), 56.4 (*N*2 aryl 4 –OCH_3_), 49.6, 48.5 (4C piperazinyl carbons), 23.6 (*N*4 aryl 4–CH_3_). Anal. Calcd for: C_27_H_27_FN_8_O_3_: C, 61.12; H, 5.13; N, 21.12; Found: C: 61.22; H: 5.14; N: 21.19.

#### 2.2.17. *N*2,*N*2-Diethyl-6-(4-(4-fluorophenyl)piperazin-1-yl)-*N*4-(4-nitrophenyl)-*s*-triazine-2,4-diamine (**4n**)

Pale yellow solid, Yield: 80%, mp 202–204°C; IR cm^−1^ (KBr): 3323 (2° –NH stretch); 1577 (–C=N ring stretch); 1312 (3° –CN stretch); 1335, 1526 (–NO stretch); 805 (CN stretch* s*-triazine). ^1^H NMR (300 MHz, DMSO-d_6_):  *δ*  2.17 (s, 6H, –CH_3_),  *δ*  3.21 (t, 4H, piperazinyl),  *δ*  3.26 (s, 4H, –CH_2_),  *δ*  3.52 (t, 4H, piperazinyl),  *δ*  6.56–8.13 (m, 8H, ArH),  *δ*  8.45 (s, 1H, NH). ^13^C NMR (75 MHz, DMSO-d_6_):  *δ*  180.3 (C6* s*-triazine, C–N piperazinyl linkage), 179.1 (C2* s*-triazine, C–N diethylamino linkage), 164.8 (C4* s*-triazine, C–N 4-nitrophenyl linkage), 150.7, 148.6, 144.1, 137.4, 129.7, 117.7, 114.9 (12C aryl carbons), 50.4, 48.7 (4C piperazinyl carbons), 43.2 (4C diethyl –CH_2_), 18.3 (6C diethyl –CH_3_). Anal. Calcd for: C_23_H_27_FN_8_O_2_: C, 59.22; H, 5.83; N, 24.02; Found: C: 59.40; H: 5.85; N: 20.07.

#### 2.2.18. *N*-(4-Bromophenyl)-4-(4-(4-fluorophenyl)piperazin-1-yl)-6-morpholino-*s*-triazin-2-amine (**4o**)

Pale yellow solid, Yield: 80%, mp 194–196°C; IR cm^−1^ (KBr): 3317 (2° –NH stretch); 1573 (–C=N ring stretch); 1121 (morpholinyl C–O–C), 810 (CN stretch* s*-triazine).^1^H NMR (75 MHz, DMSO-d_6_):  *δ*  3.23 (m, 4H, piperazinyl),  *δ*  3.46 (m, 4H, piperazinyl),  *δ*  3.51 (t, 4H, morpholinyl),  *δ*  3.83 (t, 4H, morpholinyl),  *δ*  6.39–7.44 (m, 8H, ArH),  *δ*  8.42 (s, 1H, NH). ^13^C NMR (300 MHz, DMSO-d_6_):  *δ*  181.6 (C4* s*-triazine, C–N piperazinyl linkage), 179.3 (C6* s*-triazine, C–N morpholino linkage), 158.7 (C2* s*-triazine, C–N 4-bromophenyl linkage), 153.2, 143.4, 131.5, 119.5, 117.6, 115.9 (12C aryl carbons), 65.6 (morpholino), 50.2, 49.8 (4C piperazinyl carbons), 46.5 (mopholino). Anal. Calcd for: C_23_H_25_BrFN_7_O: C, 53.70; H, 4.90; N, 19.06; Found: C: 53.50; H: 4.88; N: 19.11.

### 2.3. *In Vitro* Antimicrobial Activity

The antimicrobial potency of all the synthesized compounds was assessed* in vitro *in accordance with the guidelines of Clinical Laboratory and Standards Institute [22] against four representative Gram positive bacteria,* namely*,* Bacillus subtilis *(ATCC 6633, NCIM 2063),* Bacillus cereus *(ATCC 10876, NCIM 2156)*, Staphylococcus epidermidis *(ATCC 12228, NCIM 2493), and* Staphylococcus aureus *(NCIM 2079); Gram negative bacteria, namely,* Escherichia coli *(ATCC 8739, NCIM 2065),* Klebsiella pneumoniae* (NCIM 2706),* Proteus mirabilis *(NCIM 2241), and* Pseudomonas aeruginosa* (ATCC 19429, NCIM 2036); and two fungal strains* Candida albicans *(ATCC 2091, NCIM 3102) and* Candida tropicalis* (ATCC 13803, NCIM 3556). The microbial strains employed for the activity were procured from National Collection of Industrial Microorganisms (NCIM), Pune, India. Ciprofloxacin was used as the standard antibacterial drug and fluconazole as the standard antifungal drug.

### 2.4. Preparation of Seed Organisms

13.0 g of nutrient broth medium was suspended in 1000 mL distilled water. Then it was boiled to dissolve the medium completely and filtered from 5 *μ* sintered glass filter. A set of test tubes with nutrient broth (5 mL) was capped with cotton plugs and sterilized by autoclaving at 15 psig pressure (121°C) for 15 min. Then after cooling, a loop of organism was inoculated into liquid broth and incubated at 37 ± 1°C and used within 12 h. The inoculum size for test strain was adjusted to 10^8^ CFU/mL (colony forming unit per milliliter) by comparing the turbidity.

### 2.5. Determination of Zone of Inhibition (Disc Diffusion Method)

Petri plates containing 15 mL of Mueller Hinton agar media (Hi-Media) were used for all the bacterial strains and Sabouraud dextrose agar (SDA) media (Hi-Media) were used for the fungal strains. 0.1 mL of seed of various organisms was aseptically inoculated over the sterile solid agar medium. Whatman no. 1 filter paper discs (6 mm in diameter) impregnated with the synthesized compound (10 *μ*g/disc) dissolved in dimethyl sulfoxide (DMSO) were placed on the plates. All the sensitivity plates were then incubated at 37 ± 1°C for 24 h for bacterial strain and 72 h at 25°C for fungal strain. Ciprofloxacin (10 *μ*g/disc, Hi-Media) and fluconazole (10 *μ*g/disc, Hi-Media) were used as positive control for the assessment of antibacterial and antifungal activity, respectively. A paper disc impregnated with DMSO was used as negative control. The zone of inhibition on agar plate was measured in millimeters (mm) and the test was performed in triplicate and the average was taken as final reading (Table 2).

### 2.6. Determination of Minimum Inhibitory Concentration

Compounds that displayed favorable zone of inhibition were considered for further assessment of minimum inhibitory concentration (MIC). MIC of a compound is defined as the lowest concentration at which it completely inhibits visible growth (turbidity on liquid media). Solutions of the test compounds, ciprofloxacin and fluconazole, were prepared in DMSO at a concentration of 100 *μ*g/mL. From this stock solution, serial broth dilutions of the compounds (3.125, 6.25, 12.5, 25, 50, and 100 *μ*g/mL) were prepared to determine the MIC. All the determinations were done in triplicate and the average was taken as the final reading. The standard antibiotics, ciprofloxacin and fluconazole (100 *μ*g/mL), were used as positive controls and 100 *μ*g/mL of DMSO was used as a negative control. At the end of the incubation period (37 ± 1°C for 24 h), the MIC values were determined (Table 3).

## 3. Results and Discussion

### 3.1. Chemistry

Synthesis of the intermediates and target compounds was accomplished according to the steps illustrated in Scheme 1. The structure of the synthesized compounds was confirmed on the basis of IR, ^1^H NMR, ^13^C NMR, and elemental analysis and the spectral data are in accordance with the structures of the compounds (**4a–4o**). Only significant bands from IR are reported. The derivatives showed characteristic band in the range of 1570–1577 cm^−1^ for –C=N stretching of triazine nucleus. Further, strong absorption band in the range of 805–810 cm^−1^ indicates CN stretch of* s*-triazine moiety. A characteristic band appearing in the range of 1117–1121 cm^−1^ corresponds to the C–O–C stretching of morpholine in the IR spectra of compounds** 4b**,** 4i**, and** 4o**. The compounds also displayed –NH stretch for secondary amines that appears in the region 3315–3324 cm^−1^. The ^1^H NMR spectra of all the derivatives (**4a–4o**) displayed two triplets in the range  *δ*  2.98–3.46 and  *δ*  3.21–3.61 attributable to –CH_2_– protons of piperazine ring and each integrating for four protons except for compounds** 4e** and** 4f** that showed multiplet in the range of  *δ*  2.75–3.31. A singlet peak in the range of  *δ*  8.31–8.63 integrating for two protons corresponding to 2° –NH linkage was observed for compounds** 4a**,** 4c–4h**,** 4j**, and** 4m** while a singlet in the range  *δ*  8.42–8.52 integrating for one proton corresponding to –NH attached with* s*-triazine was observed for rest of the compounds. All the other protons belonging toaromatic ring, diethylamino, methyl, and methoxy group were seen accordingly to the expected chemical shift. The ^13^C NMR depicted the peaks of* s*-triazine nucleus that appeared at the lowest field in the range of  *δ*  179.8–183.2;  *δ*  164.6–179.7; and  *δ*  157.5–167.6 for three nonequivalent carbons. The peaks for two equivalent carbons of piperazine moiety appeared in the range of  *δ*  49.4–54.7 and  *δ*  44.6–49.8 ppm for all the compounds except** 4e** and** 4f**. All other ^13^C NMR spectra were seen according to the expected chemical shift. The elemental analysis results were within ±0.4% of thetheoretical values.

### 3.2. Antimicrobial Activity

The newly synthesized compounds (**4a**–**4o**) were evaluated for their antibacterial and antifungal activity against various Gram positive, Gram negative bacteria, and two fungal strains using agar disc diffusion method. The antimicrobial evaluation data is represented in Tables 2-3. The zone of inhibition of all the fifteen 2,4,6-trisubstituted-*s*-triazine derivatives indicates varying degree of inhibition. It was observed that compound** 4d **and** 4h **displayed equipotent antibacterial efficacy against* B. subtilis* (MIC 3.125 *μ*gmL^−1^) and* S. aureus* (MIC 6.25 *μ*gmL^−1^) under similar experimental conditions with reference to the standard drug. Considering the compounds** 4d **and** 4h **that bear* N*-methylpiperazine and* N*-(4-nitrophenyl) piperazine moiety, respectively, the compounds also possess a substituted phenyl ring at* N*4 position of* s*-triazine nucleus. Therefore, it may be suggested that irrespective of the bulkiness of piperazine ring, it is the electron withdrawing group at* para*-position of* N*4 phenyl ring that imparts the activity against Gram positive organism. This is further substantiated by the fact that the compounds substituted with electron releasing groups at* para*-position of phenyl ring (**4a**,** 4c**,** 4e**,** 4f**,** 4g**,** 4j**, and** 4m**) and compounds with morpholinyl (**4b**,** 4i**,** 4l**, and** 4o**) and dimethylamino side chain (**4k** and** 4n**) have showed either no inhibition or minimal inhibition against Gram positive organism. Alternatively, few of the aryl piperazine substituted* s*-triazine (**4g**,** 4k** and** 4n**) showed moderate to good potency against Gram negative bacteria. In particular, the* para-*fluorophenylpiperazine substituted* s*-triazine (**4n**) was found to be the most active and exhibited broad spectrum antibacterial activity. This compound was active against* S. epidermidis*,* K. Pneumoniae*, and* P. aeruginosa* with an MIC of 6.25 *μ*gmL^−1^ whereas for* E. coli *it showed an MIC of 3.125 *μ*gmL^−1^ equipotent with reference to the standard drug. The compound** 4g** was found to be active against* E. coli *and* P. aeruginosa* while compound** 4k**, wherein the* para-*fluorophenylpiperazine of** 4n** has been replaced by* para-*chlorophenylpiperazine, displayed specific activity against Gram negative strain such as* E. coli* and* K. Pneumoniae*.

Most of the compounds were incapable of showing effectiveness against the fungal strains under study. However, few compounds (**4c**,** 4g**, and** 4n**) have demonstrated antifungal potency comparable to standard fluconazole. Replacement of diethylamino group (**4n**) with* N*2* para*-methoxyphenyl ring (**4c** and** 4g**) makes the compounds more active against* C. albicans *and** 4n** was active against* C. tropicalis *that showed equipotent antifungal activity (MIC 6.25 *μ*gmL^−1^) with reference to fluconazole. Nevertheless, it was not possible to substantially correlate between the structures and antifungal activity as other compounds having phenyl ring substituted with electron releasing group were unable to elicit favourable activity (**4a**,** 4e**,** 4f**,** 4j**, and** 4m**). Among all the compounds under investigation, compound** 4g** demonstrated most significant antifungal activity against* C. albicans* (MIC 3.125 *μ*gmL^−1^, zone of inhibition 22.2 mm).

## 4. Conclusion

In summary, various 2,4,6-trisubstituted-*s*-triazine derivatives with varying degree of substitution were synthesized and evaluated for their* in vitro *antimicrobial activity. The simplicity of their preparation and better yield potentially make* s*-triazines useful and attractive antimicrobial agents. All the compounds were invariably substituted with piperazine,* N*-methylpiperazine or aryl substituted piperazine as one of the substituents of* s*-triazine scaffold. The tested compounds,** 4d**,** 4g**,** 4h**,** 4k**, and** 4n**, have shown considerable* in vitro* antibacterial efficacy at the range of 3.125–6.25 *μ*gmL^−1^ with reference to the standard drug ciprofloxacin. Irrespective of the bulkiness of piperazine ring, it is the electron withdrawing group at* para*-position of* N*4 phenyl ring that imparts good activity against Gram positive organism. From the present study, it may be concluded that the* para*-fluorophenylpiperazine derivatives of* s*-triazine (**4n**) were found to be the most active and exhibited broad spectrum antibacterial activity. Among all the compounds under investigation, compound** 4g** demonstrated most significant antifungal activity (3.125 *μ*gmL^−1^) against* C. albicans*.

## Figures and Tables

**Table 1:** 2,4,6-Trisubstituted-*s*-triazine derivatives.

Compound	R_1_	R_2_	R_3_
**4a**	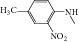	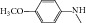	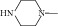
**4b**	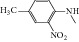	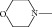	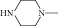
**4c**	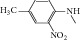	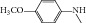	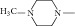
**4d**	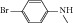	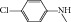	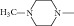
**4e**	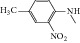	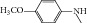	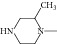
**4f**	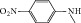	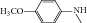	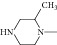
**4g**	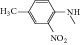	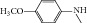	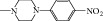
**4h **	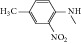	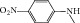	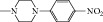
**4i**	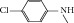	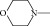	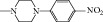
**4j**	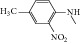	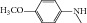	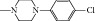
**4k**	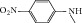	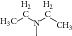	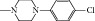
**4l**	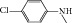	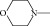	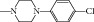
**4m**	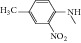	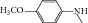	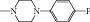
**4n**	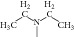	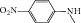	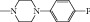
**4o**	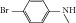	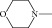	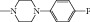

**Table 2:** Zone of inhibition (mm) of the synthesized compounds.

Compounds	Zone of inhibition (mm)^#^									
	Gram positive bacteria				Gram negative bacteria				Fungal strains	
	*B. subtilis *	*B. cereus *	*S. epidermidis *	*S. aureus *	*E. coli *	*K. pneumoniae *	*P. mirabilis *	*P. aeruginosa *	*C. albicans *	*C. tropicalis *
**4a**	18.6	16.5	—	14.0	16.0	—	—	11.8	—	16.0
**4b**	—	—	12.8	—	—	11.0	—	—	—	—
**4c**	—	14.6	—	16.8	—	18.4	18.4	17.8	21.8	19.4
**4d**	22.4	19.6	17.4	20.2	10.4	—	—	—	—	—
**4e**	—	—	14.5	—	—	15.4	16.0	—	—	—
**4f**	—	—	—	14.8		14.8	—	—	—	—
**4g**	15.3	18.4	—	—	20.0	17.0	17.6	18.2	22.2	14.8
**4h**	24.3	20.8	19.6	22.4	15.6	14.0	—	15.0	17.0	15.0
**4i**	—	15.4		—	13.5	—	12.0	—	—	—
**4j**	—	18.1	12.4	—	19.2	—	16.8	—	11.6	18.0
**4k**	11.6	—	10.6	—	20.6	19.6	17.4	18.8	—	—
**4l**	—	—	11.0	—	—	9.8	10.2	—	10.8	—
**4m**	—	17.0	—	18.0	20.0	—	12.0	—	—	—
**4n**	18.8	17.4	20.4	19.6	22.8	20.0	18.2	20.1	—	21.4
**4o**	—	11.4	—	—	13.0	—	11.2	—	—	10.2
Ciprofloxacin	25.4	23.5	21.0	26.0	25.0	24.7	20.2	21.0	NT	NT
Fluconazole	NT	NT	NT	NT	NT	NT	NT	NT	25.0	24.6

^#^Average of three determinations; NT: not tested; — indicates no inhibition.

**Table 3:** The minimum inhibitory concentration (MIC)of selected 2,4,6-trisubstituted-*s*-triazine derivatives.

Compounds	MIC (*μ*gmL^−1^)^#^									
	Gram positive bacteria				Gram negative bacteria				Fungal strains	
	*B. subtilis *	*B. cereus *	*S. epidermidis *	*S. aureus *	*E. coli *	*K. pneumoniae *	*P. mirabilis *	*P. aeruginosa *	*C. albicans *	*C. tropicalis *
**4c**	—	100	—	50	—	12.5	12.5	25	6.25	12.5
**4d**	3.125	12.5	12.5	6.25	—	—	—	—	—	—
**4g**	100	12.5	—	—	6.25	12.5	12.5	6.25	3.125	100
**4h**	3.125	12.5	12.5	6.25	50	—	—	100	25	50
**4k**	50	—	100	—	6.25	6.25	25	12.5	—	—
**4n**	12.5	12.5	6.25	12.5	3.125	6.25	12.5	6.25	50	6.25
Ciprofloxacin	3.125	6.25	6.25	6.25	3.125	3.125	6.25	6.25	NT	NT
Fluconazole	NT	NT	NT	NT	NT	NT	NT	NT	3.125	6.25

^#^Average of three determinations; NT: not tested; — indicates no inhibition.
